# Supplementary figures and images for: Induction of apoptosis in human colorectal cancer cells by nanovesicles from fingerroot (Boesenbergia rotunda (L.) Mansf.)
Source: PLoS One. 2022 Apr 4;17(4):e0266044. doi: 10.1371/journal.pone.0266044 (PMC8979466; doi:10.1371/journal.pone.0266044)

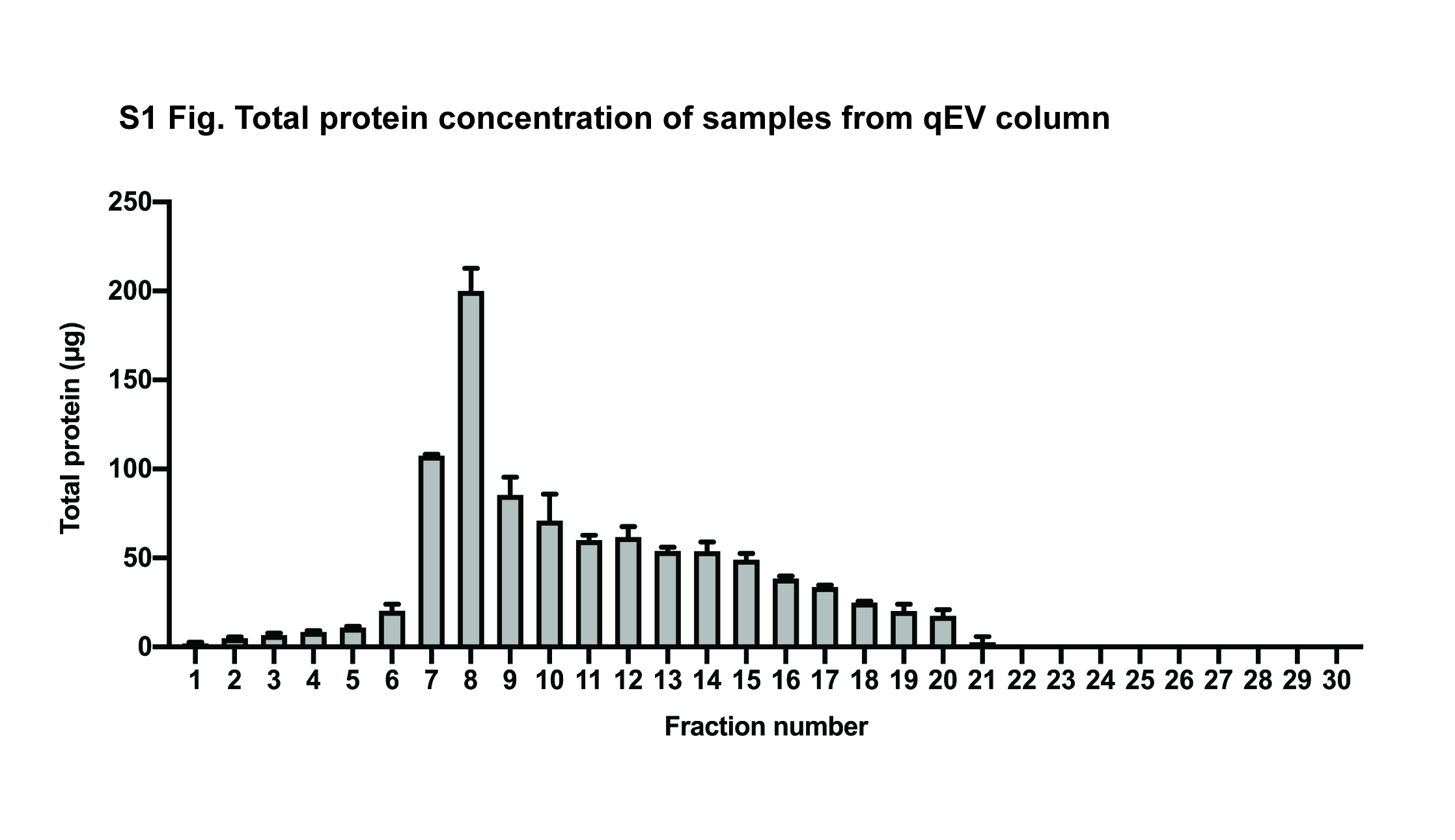

Supplement: S1 Fig — Protein concentrations of 30 fractions from qEV were determined by BCA Protein Assay. (TIF) [file pone.0266044.s001.tif]
