# Supplementary material for: Induction of apoptosis in human colorectal cancer cells by nanovesicles from fingerroot (Boesenbergia rotunda (L.) Mansf.)
Source: PLoS One. 2022 Apr 4;17(4):e0266044. doi: 10.1371/journal.pone.0266044 (PMC8979466; doi:10.1371/journal.pone.0266044)
Supplement: S1 Table — The metabolites were identified based on ChemSpider online databases, with rigorous statistical validation. (DOCX) [file pone.0266044.s002.docx]

**S1 Table. The discriminative putatively identified metabolites of FDNVs.**

| **No.** | **Name** | **Formula** | **Classes** | **Molecular weight** | **RT (min)** | **Area (AU)** |
| --- | --- | --- | --- | --- | --- | --- |
| 1 | 1-Octylthymine | C_13_H_22_N_2_O_2_ | Alkaloid | 238.17 | 16.92 | 5.50E+06 |
| 2 | 6-Methyladenosyl-l-methionine | C_16_H_26_N_6_O_5_S | Alkaloid | 414.18 | 17.52 | 7.59E+06 |
| 3 | Acetyl chloride | C_2_H_3_ClO | Organic compound | 77.99 | 0.81 | 6.54E+08 |
| 4 | Alaptide | C_9_H_14_N_2_O_2_ | Alkaloid | 182.11 | 20.29 | 1.37E+07 |
| 5 | Amide C18 | C_18_H_37_NO | Lipid | 283.28 | 23.57 | 8.98E+07 |
| 6 | Benzaldehyde | C_7_H_6_O | Organic compound | 106.04 | 10.28 | 9.05E+06 |
| 7 | Benzoylacetone | C_10_H_10_O_2_ | Organic compound | 162.07 | 7.94 | 7.10E+07 |
| 8 | Biochanin A | C_16_H_12_O_5_ | Phenolic | 284.07 | 9.18 | 6.31E+06 |
| 9 | Bromoxanide | C_19_H_18_BrF_3_N_2_O_4_ | Alkaloid | 474.04 | 0.98 | 6.90E+06 |
| 10 | Capsi-amide | C_17_H_35_NO | Lipid | 269.27 | 21.20 | 1.16E+07 |
| 11 | Choline | C_5_H_13_NO | Alkaloid | 103.10 | 0.94 | 2.46E+07 |
| 12 | Chrysin | C_15_H_10_O_4_ | Phenolic | 254.06 | 11.16 | 2.54E+07 |
| 13 | Daryamide A | C_18_H_26_N_2_O_5_ | Alkaloid | 350.18 | 19.29 | 6.44E+07 |
| 14 | Dichloroacetic acid | C_2_H_2_C_l2_O_2_ | Organic compound | 127.94 | 30.43 | 9.31E+08 |
| 15 | Dichlormid | C_8_H_12_ClNO | Organic compound | 173.06 | 0.82 | 2.27E+07 |
| 16 | DL-Arginine | C_6_H_14_N_4_O_2_ | Alkaloid | 174.11 | 0.89 | 2.33E+07 |
| 17 | DL-Atenolol | C_14_H_22_N_2_O_3_ | Alkaloid | 266.16 | 18.14 | 2.07E+07 |
| 18 | DrugRepV_4136 | C_20_H_27_N_7_O_2_ | Alkaloid | 397.22 | 20.17 | 7.40E+06 |
| 19 | Emicerfont | C_22_H_24_N_6_O_2_ | Alkaloid | 404.19 | 16.14 | 2.56E+07 |
| 20 | Esculetin | C_9_H_6_O_4_ | Phenolic | 178.02 | 21.28 | 9.13E+06 |
| 21 | Formononetin | C_16_H_12_O_4_ | Phenolic | 268.07 | 9.20 | 6.25E+06 |
| 22 | H-DL-Trp-DL-Asn-DL-Lys-DL-Lys-OH | C_27_H_42_N_8_O_6_ | Alkaloid | 574.32 | 20.03 | 2.14E+08 |
| 23 | Hexadecanamide | C_16_H_33_NO | Lipid | 255.25 | 24.09 | 2.99E+07 |
| 24 | Histidyl-prolyl-tryptophan | C_22_H_26_N_6_O_4_ | Alkaloid | 438.20 | 16.05 | 5.05E+07 |
| 25 | Hypoxanthine | C_5_H_4_N_4_O | Alkaloid | 136.04 | 1.05 | 7.96E+06 |
| 26 | Imizol | C_22_H_26_N_6_O_3_ | Alkaloid | 422.21 | 16.89 | 2.28E+08 |
| 27 | Isouron | C_10_H_17_N_3_O_2_ | Alkaloid | 211.13 | 17.45 | 8.21E+07 |
| 28 | Lauramide | C_12_H_25_NO | Lipid | 199.19 | 15.04 | 8.66E+06 |
| 29 | Levetiracetam | C_8_H_14_N_2_O_2_ | Alkaloid | 170.11 | 16.93 | 5.21E+06 |
| **No.** | **Name** | **Formula** | **Classes** | **Molecular weight** | **RT (min)** | **Area (AU)** |
| 30 | Methylimidazoleacetic acid | C_6_H_8_N_2_O_2_ | Alkaloid | 140.06 | 10.73 | 5.59E+06 |
| 31 | n6-Benzyladeninium nitrate | C_12_H_12_N_6_O_3_ | Alkaloid | 288.10 | 10.28 | 8.02E+07 |
| 32 | N-Methyl-L-histidine | C_7_H_11_N_3_O_2_ | Alkaloid | 169.09 | 13.79 | 1.04E+08 |
| 33 | N-nitroso Valsartan | C_19_H_20_N_6_O_3_ | Alkaloid | 380.16 | 14.53 | 1.31E+07 |
| 34 | Naringenin chalcone | C_15_H_12_O_5_ | Phenolic | 272.07 | 9.28 | 1.56E+08 |
| 35 | Oleamide | C_18_H_35_NO | Lipid | 281.27 | 21.82 | 6.83E+07 |
| 36 | Oseltamivir acid | C_14_H_24_N_2_O_4_ | Alkaloid | 284.17 | 16.93 | 1.35E+07 |
| 37 | Paratocarpin B | C_25_H_26_O_4_ | Phenolic | 390.18 | 21.19 | 1.24E+07 |
| 38 | Phloroglucinol | C_6_H_6_O_3_ | Phenolic | 126.03 | 9.57 | 3.32E+07 |
| 39 | Phosphanyl (phospholan-1-yl) phosphane | C_4_H_11_P_3_ | Organic compound | 152.01 | 11.43 | 2.49E+08 |
| 40 | Pilocarpine | C_11_H_16_N_2_O_2_ | Alkaloid | 208.12 | 19.62 | 3.13E+07 |
| 41 | Pinocembrin | C_15_H_12_O_4_ | Phenolic | 256.07 | 8.85 | 5.58E+07 |
| 42 | Pinostrobin | C_16_H_14_O_4_ | Phenolic | 270.09 | 9.63 | 4.01E+09 |
| 43 | Pipericine | C_22_H_41_NO | Lipid | 335.32 | 19.49 | 1.50E+07 |
| 44 | Psychrophilin E | C_25_H_24_N_4_O_4_ | Alkaloid | 444.19 | 14.60 | 5.98E+06 |
| 45 | Resveratrol | C_14_H_12_O_3_ | Phenolic | 228.08 | 11.01 | 6.08E+07 |
| 46 | Sakuranetin | C_16_H_14_O_5_ | Phenolic | 286.08 | 11.28 | 4.83E+06 |
| 47 | Simvastatin | C_25_H_38_O_5_ | Organic compound | 418.27 | 18.13 | 1.05E+07 |
| 48 | Sorbic acid | C_6_H_8_O_2_ | Lipid | 112.05 | 11.09 | 1.87E+07 |
| 49 | Terrazoanthine B | C_21_H_24_N_6_O_2_ | Alkaloid | 392.19 | 24.66 | 1.19E+07 |
| 50 | Tetracyanoindane | C_13_H_6_N4 | Alkaloid | 218.06 | 18.66 | 5.69E+06 |
| 51 | Tetraxetan | C_16_H_28_N_4_O_8_ | Alkaloid | 404.19 | 17.16 | 2.30E+07 |
| 52 | Topixantrone | C_21_H_26_N_6_O_2_ | Alkaloid | 394.21 | 16.35 | 1.01E+08 |
| 53 | Tosedostat | C_21_H_30_N_2_O_6_ | Alkaloid | 406.21 | 21.29 | 6.43E+07 |
| 54 | Triisopropanolamine | C_9_H_21_NO_3_ | Alkaloid | 191.15 | 1.01 | 5.94E+07 |
| 55 | Valerenic acid | C_15_H_22_O_2_ | Lipid | 234.16 | 15.71 | 2.68E+07 |
| 56 | Val-Trp-His | C_22_H_28_N_6_O_4_ | Alkaloid | 440.22 | 13.11 | 7.76E+07 |
| 57 | Vestitol | C_16_H_16_O_4_ | Phenolic | 272.10 | 13.46 | 1.11E+08 |
| 58 | Z-Lys(boc)-ome | C_20_H_30_N_2_O_6_ | Alkaloid | 394.21 | 17.75 | 2.87E+07 |
