# Supplementary material for: Induction of apoptosis in human colorectal cancer cells by nanovesicles from fingerroot (Boesenbergia rotunda (L.) Mansf.)
Source: PLoS One. 2022 Apr 4;17(4):e0266044. doi: 10.1371/journal.pone.0266044 (PMC8979466; doi:10.1371/journal.pone.0266044)
Supplement: S2 Table — (DOCX) [file pone.0266044.s003.docx]

**S2 Table. The minimal data set underlying the results.**

| **Fig** | **Mean** | **S.D.** | **Statistical method used** | **P value** | **Samples** |
| --- | --- | --- | --- | --- | --- |
| **Fig. 1B** | | | One-way ANOVA with Tukey's multiple comparisons test | ****P*<0.001 |  |
| Fraction 7 | 6.6×10^10^ | 2.6×10^9^ |  |  | 3 |
| Fraction 8 | 1.5×10^11^ | 7.3×10^9^ |  |  | 3 |
| Fraction 9 | 6.1×10^10^ | 2.2×10^9^ |  |  | 3 |
| **Fig. 1D** | | | One-way ANOVA with Tukey's multiple comparisons test | **P*<0.05  ***P*<0.01 |  |
| Fraction 7 | -17.4 | 3.7 |  |  | 3 |
| Fraction 8 | -26.9 | 6.1 |  |  | 3 |
| Fraction 9 | -10.6 | 5.6 |  |  | 3 |
| **Fig. 2A (FDNVs-24 h: HT-29)** | | | One-way ANOVA with Tukey's multiple comparisons test | **P*<0.05  ****P*<0.001 |  |
| 3.13 µg/ml | 99.5 | 3.6 |  |  | 3 |
| 6.25 µg/ml | 98.5 | 5.1 |  |  | 3 |
| 12.5 µg/ml | 97.3 | 5.2 |  |  | 3 |
| 25 µg/ml | 87.1 | 2.1 |  |  | 3 |
| 50 µg/ml | 61.2 | 5.8 |  |  | 3 |
| 100 µg/ml | 19.4 | 4.1 |  |  | 3 |
| **Fig. 2A (FDNVs-48 h: HT-29)** | | | One-way ANOVA with Tukey's multiple comparisons test | **P*<0.05  ****P*<0.001 |  |
| 3.13 µg/ml | 99.3 | 1.1 |  |  | 3 |
| 6.25 µg/ml | 98.1 | 3.2 |  |  | 3 |
| 12.5 µg/ml | 92.7 | 6.6 |  |  | 3 |
| 25 µg/ml | 83.7 | 9.8 |  |  | 3 |
| 50 µg/ml | 57.2 | 3.1 |  |  | 3 |
| 100 µg/ml | 1.4 | 0.8 |  |  | 3 |
| **Fig. 2A (FDNVs-72 h: HT-29)** | | | One-way ANOVA with Tukey's multiple comparisons test | ****P*<0.001 |  |
| 3.13 µg/ml | 99.7 | 2.2 |  |  | 3 |
| 6.25 µg/ml | 92.5 | 7.3 |  |  | 3 |
| 12.5 µg/ml | 89.7 | 1.3 |  |  | 3 |
| 25 µg/ml | 78.1 | 6.5 |  |  | 3 |
| 50 µg/ml | 41.7 | 7.1 |  |  | 3 |
| 100 µg/ml | 2.1 | 2.2 |  |  | 3 |

| **Fig** | **Mean** | **S.D.** | **Statistical method used** | **P value** | **Samples** |
| --- | --- | --- | --- | --- | --- |
| **Fig. 2A (Fingerroot extract-24 h: HT-29)** | | | One-way ANOVA with Tukey's multiple comparisons test | ****P*<0.001 |  |
| 3.13 µg/ml | 99.2 | 0.9 |  |  | 3 |
| 6.25 µg/ml | 97.2 | 1.3 |  |  | 3 |
| 12.5 µg/ml | 94.9 | 1.6 |  |  | 3 |
| 25 µg/ml | 82.9 | 1.2 |  |  | 3 |
| 50 µg/ml | 46.7 | 2.2 |  |  | 3 |
| 100 µg/ml | 13.6 | 5.8 |  |  | 3 |
| **Fig. 2A (Fingerroot extract-48 h: HT-29)** | | | One-way ANOVA with Tukey's multiple comparisons test | ****P*<0.001 |  |
| 3.13 µg/ml | 98.3 | 1.4 |  |  | 3 |
| 6.25 µg/ml | 97.1 | 2.7 |  |  | 3 |
| 12.5 µg/ml | 91.7 | 3.2 |  |  | 3 |
| 25 µg/ml | 79.5 | 5.6 |  |  | 3 |
| 50 µg/ml | 32.1 | 6.3 |  |  | 3 |
| 100 µg/ml | 7.4 | 3.4 |  |  | 3 |
| **Fig. 2A (Fingerroot extract-72 h: HT-29)** | | | One-way ANOVA with Tukey's multiple comparisons test | ****P*<0.001 |  |
| 3.13 µg/ml | 99.3 | 0.3 |  |  | 3 |
| 6.25 µg/ml | 98.1 | 0.5 |  |  | 3 |
| 12.5 µg/ml | 96.4 | 2.4 |  |  | 3 |
| 25 µg/ml | 77.8 | 7.7 |  |  | 3 |
| 50 µg/ml | 18.6 | 2.4 |  |  | 3 |
| 100 µg/ml | 0.9 | 0.1 |  |  | 3 |
| **Fig. 2B (FDNVs-24 h: HCT116)** | | | One-way ANOVA with Tukey's multiple comparisons test | ***P*<0.01  ****P*<0.001 |  |
| 3.13 µg/ml | 99.6 | 4.1 |  |  | 3 |
| 6.25 µg/ml | 99.5 | 6.3 |  |  | 3 |
| 12.5 µg/ml | 92.5 | 4.6 |  |  | 3 |
| 25 µg/ml | 82.4 | 6.7 |  |  | 3 |
| 50 µg/ml | 62.5 | 4.2 |  |  | 3 |
| 100 µg/ml | 13.6 | 4.5 |  |  | 3 |

| **Fig** | **Mean** | **S.D.** | **Statistical method used** | **P value** | **Samples** |
| --- | --- | --- | --- | --- | --- |
| **Fig. 2B (FDNVs-48 h: HCT116)** | | | One-way ANOVA with Tukey's multiple comparisons test | **P*<0.05  ****P*<0.001 |  |
| 3.13 µg/ml | 99.9 | 4.3 |  |  | 3 |
| 6.25 µg/ml | 95.7 | 6.6 |  |  | 3 |
| 12.5 µg/ml | 87.1 | 2.1 |  |  | 3 |
| 25 µg/ml | 75.8 | 3.2 |  |  | 3 |
| 50 µg/ml | 45.1 | 5.2 |  |  | 3 |
| 100 µg/ml | 5.4 | 3.1 |  |  | 3 |
| **Fig. 2B (FDNVs-72 h: HCT116)** | | | One-way ANOVA with Tukey's multiple comparisons test | **P*<0.05  ****P*<0.001 |  |
| 3.13 µg/ml | 99.4 | 4.5 |  |  | 3 |
| 6.25 µg/ml | 92.9 | 7.7 |  |  | 3 |
| 12.5 µg/ml | 80.5 | 8.6 |  |  | 3 |
| 25 µg/ml | 61.3 | 6.9 |  |  | 3 |
| 50 µg/ml | 27.1 | 7.4 |  |  | 3 |
| 100 µg/ml | 3.5 | 2.1 |  |  | 3 |
| **Fig. 2B (Fingerroot extract-24 h: HCT116)** | | | One-way ANOVA with Tukey's multiple comparisons test | ****P*<0.001 |  |
| 3.13 µg/ml | 99.6 | 0.2 |  |  | 3 |
| 6.25 µg/ml | 96.5 | 1.9 |  |  | 3 |
| 12.5 µg/ml | 94.9 | 0.8 |  |  | 3 |
| 25 µg/ml | 78.6 | 2.9 |  |  | 3 |
| 50 µg/ml | 36.3 | 2.3 |  |  | 3 |
| 100 µg/ml | 12.8 | 3.1 |  |  | 3 |
| **Fig. 2B (Fingerroot extract-48 h: HCT116)** | | | One-way ANOVA with Tukey's multiple comparisons test | **P*<0.05  ****P*<0.001 |  |
| 3.13 µg/ml | 98.3 | 2.1 |  |  | 3 |
| 6.25 µg/ml | 97.7 | 4.1 |  |  | 3 |
| 12.5 µg/ml | 90.5 | 2.9 |  |  | 3 |
| 25 µg/ml | 75.7 | 1.6 |  |  | 3 |
| 50 µg/ml | 25.1 | 6.1 |  |  | 3 |
| 100 µg/ml | 6.6 | 0.1 |  |  | 3 |

| **Fig** | **Mean** | **S.D.** | **Statistical method used** | **P value** | **Samples** |
| --- | --- | --- | --- | --- | --- |
| **Fig. 2B (Fingerroot extract-72 h: HCT116)** | | | One-way ANOVA with Tukey's multiple comparisons test | **P*<0.05  ****P*<0.001 |  |
| 3.13 µg/ml | 98.2 | 0.5 |  |  | 3 |
| 6.25 µg/ml | 96.7 | 3.2 |  |  | 3 |
| 12.5 µg/ml | 87.6 | 4.9 |  |  | 3 |
| 25 µg/ml | 61.1 | 5.6 |  |  | 3 |
| 50 µg/ml | 10.6 | 5.5 |  |  | 3 |
| 100 µg/ml | 0.8 | 0.7 |  |  | 3 |
| **Fig. 2C (FDNVs-24 h: CCD 841 CoN)** | | | One-way ANOVA with Tukey's multiple comparisons test | - |  |
| 3.13 µg/ml | 99.3 | 5.6 |  |  | 3 |
| 6.25 µg/ml | 99.5 | 4.5 |  |  | 3 |
| 12.5 µg/ml | 98.7 | 4.5 |  |  | 3 |
| 25 µg/ml | 98.5 | 3.2 |  |  | 3 |
| 50 µg/ml | 93.5 | 5.9 |  |  | 3 |
| 100 µg/ml | 90.2 | 4.2 |  |  | 3 |
| **Fig. 2C (FDNVs-48 h: CCD 841 CoN)** | | | One-way ANOVA with Tukey's multiple comparisons test | **-** |  |
| 3.13 µg/ml | 99.7 | 4.5 |  |  | 3 |
| 6.25 µg/ml | 99.1 | 7.8 |  |  | 3 |
| 12.5 µg/ml | 96.2 | 9.6 |  |  | 3 |
| 25 µg/ml | 95.4 | 8.9 |  |  | 3 |
| 50 µg/ml | 91.4 | 2.8 |  |  | 3 |
| 100 µg/ml | 85.3 | 6.6 |  |  | 3 |
| **Fig. 2C (FDNVs-72 h: CCD 841 CoN)** | | | One-way ANOVA with Tukey's multiple comparisons test | ***P*<0.01 |  |
| 3.13 µg/ml | 99.6 | 2.2 |  |  | 3 |
| 6.25 µg/ml | 98.7 | 6.7 |  |  | 3 |
| 12.5 µg/ml | 95.3 | 8.4 |  |  | 3 |
| 25 µg/ml | 91.5 | 0.5 |  |  | 3 |
| 50 µg/ml | 88.6 | 5.5 |  |  | 3 |
| **Fig** | **Mean** | **S.D.** | **Statistical method used** | **P value** | **Samples** |
| 100 µg/ml | 83.2 | 6.1 |  |  | 3 |
| **Fig. 2C (Fingerroot extract-24 h: CCD 841 CoN)** | | | One-way ANOVA with Tukey's multiple comparisons test | ****P*<0.001 |  |
| 3.13 µg/ml | 98.9 | 1.6 |  |  | 3 |
| 6.25 µg/ml | 94.8 | 1.6 |  |  | 3 |
| 12.5 µg/ml | 92.9 | 1.8 |  |  | 3 |
| 25 µg/ml | 79.7 | 5.3 |  |  | 3 |
| 50 µg/ml | 42.8 | 3.1 |  |  | 3 |
| 100 µg/ml | 8.8 | 6.2 |  |  | 3 |
| **Fig. 2C (Fingerroot extract-48 h: CCD 841 CoN)** | | | One-way ANOVA with Tukey's multiple comparisons test | ***P*<0.01  ****P*<0.001 |  |
| 3.13 µg/ml | 98.1 | 1.1 |  |  | 3 |
| 6.25 µg/ml | 93.4 | 0.6 |  |  | 3 |
| 12.5 µg/ml | 86.8 | 3.9 |  |  | 3 |
| 25 µg/ml | 72.1 | 2.5 |  |  | 3 |
| 50 µg/ml | 28.4 | 0.7 |  |  | 3 |
| 100 µg/ml | 5.1 | 7.5 |  |  | 3 |
| **Fig. 2C (Fingerroot extract-72 h: CCD 841 CoN)** | | | One-way ANOVA with Tukey's multiple comparisons test | ***P*<0.01  ****P*<0.001 |  |
| 3.13 µg/ml | 98.8 | 0.6 |  |  | 3 |
| 6.25 µg/ml | 94.9 | 1.3 |  |  | 3 |
| 12.5 µg/ml | 83.6 | 3.5 |  |  | 3 |
| 25 µg/ml | 51.4 | 5.7 |  |  | 3 |
| 50 µg/ml | 18.8 | 9.1 |  |  | 3 |
| 100 µg/ml | 0.4 | 0.1 |  |  | 3 |
| **Fig. 3D (12.5 µg/ml)** | | | One-way ANOVA with Tukey's multiple comparisons test | ****P*<0.001 |  |
| HT-29 | 31.1 | 2.9 |  |  | 3 |
| HCT116 | 11.3 | 0.3 |  |  | 3 |
| CCD 841 CoN | 1.1 | 0.1 |  |  | 3 |

| **Fig** | **Mean** | **S.D.** | **Statistical method used** | **P value** | **Samples** |
| --- | --- | --- | --- | --- | --- |
| **Fig. 3D (25 µg/ml)** | | | One-way ANOVA with Tukey's multiple comparisons test | ****P*<0.001 |  |
| HT-29 | 44.6 | 0.5 |  |  | 3 |
| HCT116 | 31.9 | 2.3 |  |  | 3 |
| CCD 841 CoN | 3.4 | 0.1 |  |  | 3 |
| **Fig. 4A (HT-29)** | | | One-way ANOVA with Tukey's multiple comparisons test | ****P*<0.001 |  |
| Chlorpromazine | 93.7 | 13.9 |  |  | 3 |
| Amiloride | 84.1 | 8.9 |  |  | 3 |
| Filipin | 33.1 | 7.5 |  |  | 3 |
| **Fig. 4A (HCT116)** | | |  |  |  |
| Chlorpromazine | 90.4 | 8.9 |  |  | 3 |
| Amiloride | 85.1 | 13.1 |  |  | 3 |
| Filipin | 31.6 | 9.6 |  |  | 3 |
| **Fig. 4B (HT-29)** | | | One-way ANOVA with Tukey's multiple comparisons test | ****P*<0.001 |  |
| Cytochalasin D | 10.9 | 8.4 |  |  | 3 |
| **Fig. 4B (HCT116)** | | |  |  |  |
| Cytochalasin D | 16.8 | 12.1 |  |  | 3 |
| **Fig. 5A (HT-29: Viable cells**) | | | One-way ANOVA with Tukey's multiple comparisons test | **P*<0.05  ****P*<0.001 |  |
| Untreated | 91.8 | 0.2 |  |  | 3 |
| 25 µg/ml | 72.9 | 7.1 |  |  | 3 |
| 50 µg/ml | 44.5 | 10.5 |  |  | 3 |
| 100 µg/ml | 10.5 | 7.4 |  |  | 3 |
| 5% DMSO | 32.7 | 2.7 |  |  | 3 |
| **Fig. 5A (HT-29: Early apoptosis**) | | | One-way ANOVA with Tukey's multiple comparisons test | **P*<0.05  ****P*<0.001 |  |
| Untreated | 2.9 | 1.1 |  |  | 3 |
| 25 µg/ml | 6.4 | 1.2 |  |  | 3 |
| 50 µg/ml | 12.2 | 2.4 |  |  | 3 |
| 100 µg/ml | 18.1 | 2.4 |  |  | 3 |
| 5% DMSO | 25.5 | 1.3 |  |  | 3 |
| **Fig. 5A (HT-29: Late apoptosis**) | | | One-way ANOVA with Tukey's multiple comparisons test | ***P*<0.01  ****P*<0.001 |  |
| Untreated | 4.8 | 0.1 |  |  | 3 |
| 25 µg/ml | 18.7 | 6.4 |  |  | 3 |

| **Fig** | **Mean** | **S.D.** | **Statistical method used** | **P value** | **Samples** |
| --- | --- | --- | --- | --- | --- |
| 50 µg/ml | 35.8 | 3.3 |  |  | 3 |
| 100 µg/ml | 61.3 | 8.4 |  |  | 3 |
| 5% DMSO | 36.3 | 8.1 |  |  | 3 |
| **Fig. 5A (HT-29: Necrosis**) | | | One-way ANOVA with Tukey's multiple comparisons test | **P*<0.05 |  |
| Untreated | 0.3 | 0.2 |  |  | 3 |
| 25 µg/ml | 1.8 | 1.4 |  |  | 3 |
| 50 µg/ml | 7.4 | 6.1 |  |  | 3 |
| 100 µg/ml | 9.8 | 0.6 |  |  | 3 |
| 5% DMSO | 5.4 | 4.1 |  |  | 3 |
| **Fig. 5B (HCT116: Viable cells**) | | | One-way ANOVA with Tukey's multiple comparisons test | **P*<0.05  ****P*<0.001 |  |
| Untreated | 93.2 | 3.1 |  |  | 3 |
| 25 µg/ml | 78.5 | 4.6 |  |  | 3 |
| 50 µg/ml | 64.5 | 3.8 |  |  | 3 |
| 100 µg/ml | 6.4 | 6.1 |  |  | 3 |
| 5% DMSO | 14.7 | 1.2 |  |  | 3 |
| **Fig. 5B (HCT116: Early apoptosis**) | | | One-way ANOVA with Tukey's multiple comparisons test | **P*<0.05  ***P*<0.01  ****P*<0.001 |  |
| Untreated | 1.4 | 0.7 |  |  | 3 |
| 25 µg/ml | 5.1 | 4.7 |  |  | 3 |
| 50 µg/ml | 11.3 | 5.6 |  |  | 3 |
| 100 µg/ml | 19.8 | 3.1 |  |  | 3 |
| 5% DMSO | 24.1 | 0.8 |  |  | 3 |
| **Fig. 5B (HCT116: Late apoptosis**) | | | One-way ANOVA with Tukey's multiple comparisons test | **P*<0.05  ****P*<0.001 |  |
| Untreated | 4.1 | 2.4 |  |  | 3 |
| 25 µg/ml | 10.7 | 3.5 |  |  | 3 |
| 50 µg/ml | 17.1 | 6.6 |  |  | 3 |
| 100 µg/ml | 67.7 | 6.1 |  |  | 3 |
| 5% DMSO | 56.7 | 1.1 |  |  | 3 |
| **Fig. 5B (HCT116: Necrosis**) | | | One-way ANOVA with Tukey's multiple comparisons test | - |  |
| Untreated | 1.2 | 0.2 |  |  | 3 |
| 25 µg/ml | 5.5 | 6.8 |  |  | 3 |

| **Fig** | **Mean** | **S.D.** | **Statistical method used** | **P value** | **Samples** |
| --- | --- | --- | --- | --- | --- |
| 50 µg/ml | 7.1 | 5.2 |  |  | 3 |
| 100 µg/ml | 5.9 | 0.5 |  |  | 3 |
| 5% DMSO | 4.4 | 0.4 |  |  | 3 |
| **Fig. 5C (CCD 841 CoN: Viable cells**) | | | One-way ANOVA with Tukey's multiple comparisons test | ****P*<0.001 |  |
| Untreated | 92.6 | 1.2 |  |  | 3 |
| 25 µg/ml | 92.9 | 1.2 |  |  | 3 |
| 50 µg/ml | 92.9 | 1.4 |  |  | 3 |
| 100 µg/ml | 91.1 | 1.8 |  |  | 3 |
| 5% DMSO | 65.4 | 5.1 |  |  | 3 |
| **Fig. 5C (CCD 841 CoN: Early apoptosis**) | | | One-way ANOVA with Tukey's multiple comparisons test | ****P*<0.001 |  |
| Untreated | 3.9 | 2.5 |  |  | 3 |
| 25 µg/ml | 2.7 | 2.9 |  |  | 3 |
| 50 µg/ml | 3.1 | 3.6 |  |  | 3 |
| 100 µg/ml | 2.9 | 4.2 |  |  | 3 |
| 5% DMSO | 32.2 | 5.1 |  |  | 3 |
| **Fig. 5C (CCD 841 CoN: Late apoptosis**) | | | One-way ANOVA with Tukey's multiple comparisons test | - |  |
| Untreated | 1.8 | 1.4 |  |  | 3 |
| 25 µg/ml | 1.4 | 0.2 |  |  | 3 |
| 50 µg/ml | 1.1 | 0.2 |  |  | 3 |
| 100 µg/ml | 1.1 | 1.2 |  |  | 3 |
| 5% DMSO | 0.3 | 0.1 |  |  | 3 |
| **Fig. 5C (CCD 841 CoN: Necrosis**) | | | One-way ANOVA with Tukey's multiple comparisons test | - |  |
| Untreated | 1.5 | 1.7 |  |  | 3 |
| 25 µg/ml | 2.8 | 1.9 |  |  | 3 |
| 50 µg/ml | 2.9 | 2.4 |  |  | 3 |
| 100 µg/ml | 4.9 | 3.8 |  |  | 3 |
| 5% DMSO | 0.1 | 0.1 |  |  | 3 |

| **Fig** | **Mean** | **S.D.** | **Statistical method used** | **P value** | **Samples** |
| --- | --- | --- | --- | --- | --- |
| **Fig. 6A (HT-29: Caspase 3**) | | | One-way ANOVA with Tukey's multiple comparisons test | ***P*<0.01  ****P*<0.001 |  |
| 6.25 µg/ml | 1.07 | 0.02 |  |  | 3 |
| 12.5 µg/ml | 1.21 | 0.07 |  |  | 3 |
| 25 µg/ml | 1.46 | 0.11 |  |  | 3 |
| **Fig. 6A (HT-29: Caspase 9**) | | | One-way ANOVA with Tukey's multiple comparisons test | ****P*<0.001 |  |
| 6.25 µg/ml | 1.07 | 0.04 |  |  | 3 |
| 12.5 µg/ml | 1.52 | 0.14 |  |  | 3 |
| 25 µg/ml | 2.09 | 0.12 |  |  | 3 |
| **Fig. 6A (HT-29: Bax**) | | | One-way ANOVA with Tukey's multiple comparisons test | ****P*<0.001 |  |
| 6.25 µg/ml | 1.07 | 0.04 |  |  | 3 |
| 12.5 µg/ml | 1.15 | 0.05 |  |  | 3 |
| 25 µg/ml | 1.34 | 0.09 |  |  | 3 |
| **Fig. 6A (HT-29: Bcl-2**) | | | One-way ANOVA with Tukey's multiple comparisons test | ***P*<0.01  ****P*<0.001 |  |
| 6.25 µg/ml | 0.97 | 0.02 |  |  | 3 |
| 12.5 µg/ml | 0.76 | 0.09 |  |  | 3 |
| 25 µg/ml | 0.61 | 0.16 |  |  | 3 |
| **Fig. 6B (HCT116: Caspase 3**) | | | One-way ANOVA with Tukey's multiple comparisons test | ***P*<0.01  ****P*<0.001 |  |
| 6.25 µg/ml | 1.15 | 0.14 |  |  | 3 |
| 12.5 µg/ml | 1.41 | 0.17 |  |  | 3 |
| 25 µg/ml | 1.52 | 0.18 |  |  | 3 |
| **Fig. 6B (HCT116: Caspase 9**) | | | One-way ANOVA with Tukey's multiple comparisons test | ***P*<0.01  ****P*<0.001 |  |
| 6.25 µg/ml | 1.22 | 0.19 |  |  | 3 |
| 12.5 µg/ml | 1.42 | 0.26 |  |  | 3 |
| 25 µg/ml | 2.32 | 0.31 |  |  | 3 |
| **Fig. 6B (HCT116: Bax**) | | | One-way ANOVA with Tukey's multiple comparisons test | **P*<0.05 |  |
| 6.25 µg/ml | 1.01 | 0.02 |  |  | 3 |
| 12.5 µg/ml | 1.17 | 0.06 |  |  | 3 |
| 25 µg/ml | 1.37 | 0.07 |  |  | 3 |
| **Fig. 6B (HCT116: Bcl-2**) | | | One-way ANOVA with Tukey's multiple comparisons test | **P*<0.05 |  |
| 6.25 µg/ml | 0.95 | 0.03 |  |  | 3 |
| 12.5 µg/ml | 0.85 | 0.11 |  |  | 3 |

| **Fig** | **Mean** | **S.D.** | **Statistical method used** | **P value** | **Samples** |
| --- | --- | --- | --- | --- | --- |
| 25 µg/ml | 0.67 | 0.09 |  |  | 3 |
| **Fig. 7A (HT-29: ROS**) | | | One-way ANOVA with Tukey's multiple comparisons test | **P*<0.05  ***P*<0.01  ****P*<0.001 |  |
| 12.5 µg/ml | 1.2 | 0.18 |  |  | 3 |
| 25 µg/ml | 1.4 | 0.04 |  |  | 3 |
| 50 µg/ml | 1.4 | 0.09 |  |  | 3 |
| 200 µM H_2_O_2_ | 1.3 | 0.13 |  |  | 3 |
| **Fig. 7A (HT-29: GSH**) | | | One-way ANOVA with Tukey's multiple comparisons test | **P*<0.05  ****P*<0.001 |  |
| Untreated | 73.8 | 3.1 |  |  | 3 |
| 12.5 µg/ml | 69.2 | 0.6 |  |  | 3 |
| 25 µg/ml | 65.5 | 3.2 |  |  | 3 |
| 50 µg/ml | 34.3 | 7.2 |  |  | 3 |
| 200 µM H_2_O_2_ | 18.1 | 1.9 |  |  | 3 |
| **Fig. 7B (HCT116: ROS**) | | | One-way ANOVA with Tukey's multiple comparisons test | **P*<0.05  ***P*<0.01  ****P*<0.001 |  |
| 12.5 µg/ml | 1.5 | 0.22 |  |  | 3 |
| 25 µg/ml | 1.7 | 0.14 |  |  | 3 |
| 50 µg/ml | 1.8 | 0.21 |  |  | 3 |
| 200 µM H_2_O_2_ | 1.4 | 0.09 |  |  | 3 |
| **Fig. 7B (HCT116: GSH**) | | | One-way ANOVA with Tukey's multiple comparisons test | ***P*<0.01  ****P*<0.001 |  |
| Untreated | 80.8 | 3.9 |  |  | 3 |
| 12.5 µg/ml | 66.4 | 3.9 |  |  | 3 |
| 25 µg/ml | 60.8 | 1.3 |  |  | 3 |
| 50 µg/ml | 47.8 | 2.7 |  |  | 3 |
| 200 µM H_2_O_2_ | 35.3 | 1.3 |  |  | 3 |
| **Fig. 7C (CCD 841 CoN: ROS**) | | | One-way ANOVA with Tukey's multiple comparisons test | **P*<0.05 |  |
| 12.5 µg/ml | 1 | 0.03 |  |  | 3 |
| 25 µg/ml | 1 | 0.02 |  |  | 3 |
| 50 µg/ml | 1 | 0.02 |  |  | 3 |
| 200 µM H_2_O_2_ | 1.2 | 0.03 |  |  | 3 |
| **Fig. 7C (CCD 841 CoN: GSH**) | | | One-way ANOVA with Tukey's multiple comparisons test | ***P*<0.01 |  |
| Untreated | 64.5 | 1.9 |  |  | 3 |
| 12.5 µg/ml | 62.7 | 1.9 |  |  | 3 |

| **Fig** | **Mean** | **S.D.** | **Statistical method used** | **P value** | **Samples** |
| --- | --- | --- | --- | --- | --- |
| 25 µg/ml | 63.2 | 1.3 |  |  | 3 |
| 50 µg/ml | 63.5 | 2.6 |  |  | 3 |
| 200 µM H_2_O_2_ | 50.1 | 1.3 |  |  | 3 |
